# Antiparasitic agents produced by microorganisms

**Published:** 2004-06-01

**Authors:** Kazuro Shiomi, Satoshi Ōmura

**Affiliations:** *) School of Pharmaceutical Sciences, Kitasato University, 5-9-1 Shirokane, Minato-ku, Tokyo 108-8641, Japan; **) Kitasato Institute for Life Sciences and Graduate School of Infection Control Sciences, Kitasato University and The Kitasato Institute, 5-9-1 Shirokane, Minato-ku, Tokyo 108-8641, Japan

**Keywords:** Antibiotics, antiparasite, antiprotozoal, anthelmintic

## Abstract

Microorganisms produce many useful antiparasitic agents. Antiparasitic activities and biochemical targets of antiprotozoal and anthelmintic antibiotics are reviewed. Antimalarial apicidin, thiolactomycin, fosmidomycin, and borrelidin, antitrypanosomal ascofuranone, and nematocidal emodepside, 2-deoxoparaherquamide A, and nafuredin are recently discovered antibiotics, and they have potential as useful drugs.

## Introduction

**Fig. 1:**
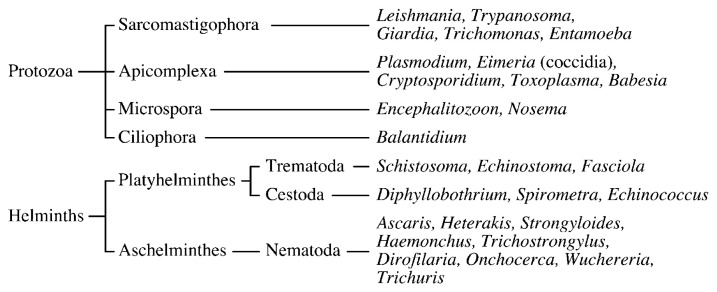
Classification of parasites.

Parasites are organisms that range from unicellular eukaryotes (protozoa) to multicellular eukaryotes (helminths) (Fig. 1).

Many antiparasitic drugs are compounds of natural origin or their derivatives. Quinine is an alkaloid derived from the bark of *Cinchona* spp., and artemisinin is a sesquiterpene lactone isolated from the Chinese medicinal herb *Artemisia annua*. Both are used as antimalarials. *α*-Santonin is a sesquiterpene isolated from the unexpanded flower heads of *Artemisia* spp., and kainic acid is an amino acid isolated from the red alga *Digenea simplex*. They are used as nematocidals. There are also antiparasitic drugs derived from microorganisms.

Since Alexander Fleming discovered the antibacterial activity of penicillin in 1928, more than 20,000 antibiotics have been reported.1) Most of them show antibacterial activity, but some have other interesting activities, for example, antifungal, antiviral, anticancer, herbicidal, insecticidal, or antiparasitic activities. Among them, about 600 have been reported to show antiparasitic activities.2) However, most compounds are cytotoxic, and only a few compounds are used practically. These include the antiprotozoal antibiotics, tetracyclines, polyethers, spiramycin, acetylspiramycin, paromomycin, clindamycin, amphotericin B, trichomycin, and fumagillin, the anthelmic antibiotics, ivermectin and paromomycin, and the veterinary nematocidal antibiotics, milbemycin D, destomycin A, and hygromycin B. Their activities and the microorganisms that produce them are summarized in Table I.

In this paper, we review antiparasitic antibiotics produced by microorganisms. In addition to those used as antiparasitic antibiotics, we review antibiotics that are under development and new antibiotics that have interesting activities.

## Antiprotozoals

### (a) Antimalarials

**Fig. 2:**
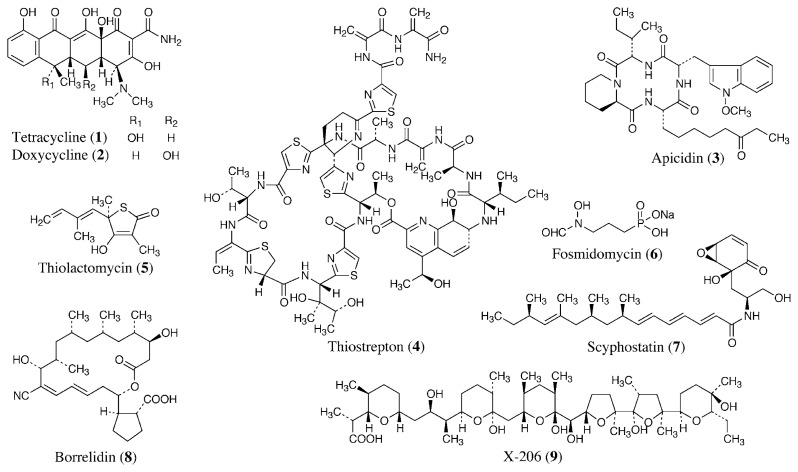
Antimalarial antibiotics.

Tetracyclines (**1**, **2**, Fig. 2) are broad-spectrum antibacterial agents active against gram-positive and -negative bacteria, mycoplasmas, rickettsiae, and chlamydiae. They are polyketide antibiotics isolated from culture broths of the actinomycetes *Streptomyces* spp.3)–5) or their semi-synthetic derivatives. Tetracyclines are also used as supplements to quinine in the treatment of malaria caused by *Plasmodium falciparum*, when resistance to quinine has been reported in patients and are also effective for prophylaxis of multi-drug resistant *P. falciparum*.6) They have potent but slow action against the asexual blood stages of all plasmodial species. They are also active against the primary intrahepatic stages of *P. falciparum*. In addition, tetracyclines have been used to treat infections with *Entamoeba histolytica*, *Giardia lamblia*, *Leishmania major*, *Trichomonas vaginalis*, and *Toxoplasma gondii*.7) Tetracycline treatment of filarial nematode-infected animals results in reduced numbers of adult worms and microfilariae, suggesting that tetracyclines may be beneficial for treatment of humans infected with filarial nematodes.8)

A chloroplast-like organelle (apicoplast) is present in many species of the phylum Apicomplexa, containing *Plasmodium*, *Toxoplasma*, *Eimeria*, and *Cryptosporidium*. The organelle is essential for blood-stage development of *Plasmodium* and the formation of the parasitophorous vacuole of *Toxoplasma*. Tetracyclines are protein synthesis inhibitors that block binding of aminoacyl-tRNA to the A site of 16S rRNA on the 30S subunit of the prokaryotic ribosome.9) Malarial parasites have two extrachromosomal DNAs with prokaryotic organelle-like characteristics, apicoplast and mitochondrial DNA. Tetracyclines are suggested to inhibit apicoplast and mitochondrial protein synthesis of *Plasmodium*.10)

Apicidin (**3**) is a cyclotetrapeptide produced by the fungus *Fusarium pallidoroseum*. It exhibits potent, broad-spectrum antiprotozoal activity *in vitro* against Apicomplexan parasites. It is orally and parenterally active against *P. berghei* malaria in mice.11) Its antiparasitic activity appears to be due to low nanomolar inhibition of Apicomplexan histone deacetylase, which induces hyperacetylation of histones in treated parasites. Antimalarial activities have also been reported in other peptide antibiotics, such as cyclic peptide (takaokamycin),12) cyclic depsipeptides (enniatins),13) linear peptides (leucinostatin A12) and efrapeptins14)), and peptaibols (antiamebin I and zervamicins).14)

A thiazole-containing cyclic depsipeptide, thiostrepton (**4**) produced by *Streptomyces azureus*, eliminates infection with erythrocytic forms of *Plasmodium berghei* in mice. It interacts with the GTPase binding domain of the apicoplast large subunit rRNA and inhibits protein synthesis, as it does in bacteria. 15)

It has recently been demonstrated that type II fatty acid biosynthesis occurs in the apicoplast, and an inhibitor of this pathway, thiolactomycin (**5**), produced by the actinomycete *Nocardia* sp., restricts the growth of *P. falciparum* and *Toxoplasma gondii*.16) The type II pathway is common to plant chloroplasts and bacteria but distinct from the type I pathway of mammals. Furthermore, *Trypanosoma brucei*, which belongs to the phylum Sarcomastigophora, also uses type II fatty acid biosynthesis for myristate synthesis, and is susceptible to thiolactomycin.

In some eubacteria, algae, and plants, the 1-deoxy-d-xylulose 5-phosphate (DOXP) pathway was described as an alternative nonmevalonate pathway for the early steps in the biosynthesis of isoprenoids. This pathway is present in *Plasmodium*, and its DOXP reductoisomerase is inhibited by fosmidomycin (**6**) produced by *Streptomyces lavendulae*.17) Treatment with fosmidomycin for acute uncomplicated *P. falciparum* infection has been effective in clinical trials.18)

*P. falciparum* phospholipase C hydrolyzes sphingomyelin and lysocholine phospholipids, and is inhibited by a mammalian neutral sphingomyelinase inhibitor, scyphostatin (**7**), produced by the fungus *Trichopeziza mollissima*.19) It also inhibits the intraerythrocytic proliferation of *P. falciparum*.

Borrelidin (**8**) is produced by *Streptomyces* spp.,20) and it is a nonglycosidic 18-membered ring macrolide with a side chain of cyclopentanecarboxylic acid. It has various biological activities, such as antimicrobial, antiviral, herbicidal, insecticidal, and antitumor activities. The activities may be due to the inhibition of threonyl-tRNA synthetase and the activation of caspase-3 and caspase-8.21) Recently, our group found that borrelidin shows excellent antimalarial activity against both chloroquine-sensitive and -resistant *P. falciparum in vitro*.22) It also has antimalarial activity against chloroquine-sensitive and -resistant strains in mice. The effective dose is lower than that of artemether, artesunate, and chloroquine in each experiment.

In the same screening, we found that polyether antibiotics have a high selective inhibitory effect against *P. falciparum in vitro*. Among these polyethers, X-206 (**9**), produced by an actinomycete,23) shows the highest selectivity and potency.24) Although, it has potent *in vivo* antimalarial activity in mice, the therapeutic window is narrow compared with its selective toxicity *in vitro*.

### (b) Anticoccidials, anticryptosporidials & antitoxoplasmals

**Fig. 3:**
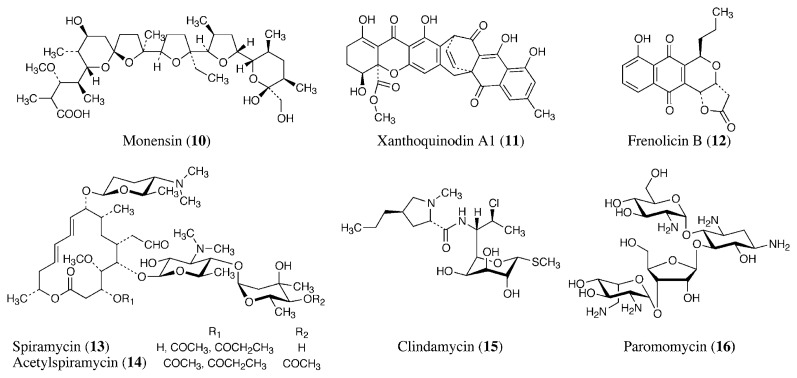
Anticoccidial, anticryptosporidial, and antitoxoplasmal antibiotics.

Polyether antibiotics such as monensin (**10**, Fig. 3),25) lasalocid A, and salinomycin are produced by actinomycetes and are used as feed additives for the prevention of poultry coccidiosis. They are ionophore antibiotics that increase the influx of monovalent cations through the membrane, causing swelling and death of the parasites. The development of resistance to anticoccidial agents is now posing similar problems to those encountered with malaria.26) No active member from a new class of chemical substances had been developed for more than 10 years, so our group screened anticoccidial antibiotics using monensin-resistant *Eimeria tenella in vitro*. As a result, we isolated several new antibiotics, including xanthoquinodins,27) diolmycins,28) hynapenes,29) arohynapenes,30) cytosaminomycins,31),32) and fudecalone.33) Xanthoquinodin A1 (**11**), produced by a fungus (*Humicola* sp.), showed anticoccidial activity against a monensin-resistant strain *in vivo*. Frenolicin B (**12**) was originally isolated as an antimycoplasmal antibiotic from *Streptomyces roseofulvus* by our group,34) and later was revealed to show potent anticoccidial activity *in vivo*.35)

Spiramycin (**13**) is a 16-membered macrolide produced by *Streptomyces ambofaciens* and used as an antibacterial in the veterinary field.36) It is also effective in treating patients with cryptosporidiosis.37) Its acetylated derivative, acetylspiramycin (**14**), is used as an antibacterial antibiotic for humans, and also used to treat toxoplasmosis.38) Clindamycin (**15**) is a semi-synthetic antibiotic prepared from lincomycin, which is produced by *Streptomyces lincolnensis*.39) It is effective against toxoplasmosis and babesiosis.6)

Spiramycins and clindamycin inhibit protein synthesis by binding to 23S rRNA on the 50S subunit of the prokaryotic ribosome. They bind adjacent to the peptidyltransferase center of the 50S subunit, and their binding sites partially overlap.9) They do not inhibit chromosomal or mitochondrial ribosomes.40) Clindamycin-resistant mutants of *Toxoplasma gondii* have a point mutation in the apicoplast large-subunit rRNA, and the position of the mutation corresponds to the position in *Escherichia coli* predicted to bind clindamycin. The mutants show cross-resistance to macrolides. Therefore, spiramycins and clindamycin are believed to target the apicoplast ribosome.41)

Paromomycin (**16**), produced by *Streptomyces rimosus*, is a member of the aminoglycoside antibiotics. 42) It binds to 16S rRNA on the 30S subunit of the ribosome and inhibits protein synthesis by increasing the affinity of the A-site for tRNA, inducing errors in translation. 43) Paromomycin, an anthelmintic for cestode infection, is also used for cryptosporidiosis, amebiasis, and giardiasis.44)

### (c) Antileishmanial & antitrypanosomals

**Fig. 4:**
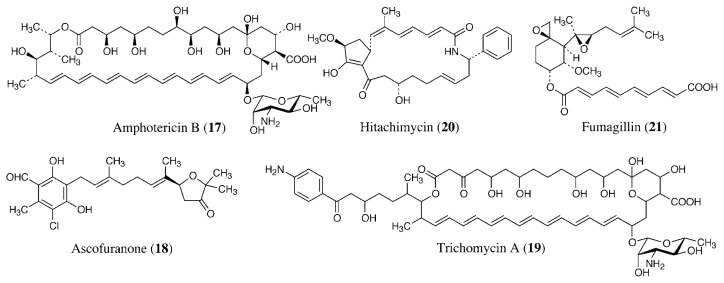
Antileishmanial, antitrypanosomal, antitrichomonal, and antimicrosporidial antibiotics.

Amphotericin B (**17**, Fig. 4), produced by *Streptomyces nodosus*, is a widely used 38-membered ring heptaene macrolide with antifungal properties.45) Amphotericin B or its lipid formulations (liposomal amphotericin B) are also used for visceral leishmaniasis (kala-azar) and mucocutaneous leishmaniasis unresponsive to pentavalent antimony compounds.6),46) The cell membrane of *Leishmania* parasites are composed of ergosterol-related sterols similar to those of fungi. Therefore, amphotericin B can bind to the membrane and kill the parasites. Although the growth of *Leishmania* parasites is susceptible to azoles and other ergosterol biosynthesis inhibitors, they can survive with greatly altered sterol profiles induced by continuous treatment with low concentration of the inhibitors, and they also have some ability to utilize and metabolize host sterol.16) Therefore, ergosterol biosynthesis inhibitors are not used in practice for leishmaniasis but are still in clinical studies.

Ascofuranone (**18**) was originally isolated from the culture broth of the fungus *Ascochyta viciae* as an antibiotic that reduced serum lipid levels of rats.47) Recently, it was revealed to inhibit the trypanosome alternative oxidase, which is the terminal oxidase of the respiratory chain of long slender bloodstream forms of *Trypanosoma brucei*.48) Ascofuranone shows good therapeutic effects for African trypanosomiasis in mice.

### (d) Antitrichomonals

Trichomycin is used as an antifungal and antitrichomonal antibiotic. It is a mixture of polyene macrolides produced by *Streptomyces hachijoensis*.49) The major component is a 38-membered ring heptaene macrolide, trichomycin A (**19**).50) Hitachimycin (**20**) isolated by our group is a 19-membered ring lactam produced by *Streptomyces* sp.51) It shows antitrichomonal and antitumor activities.

### (e) Antimicrosporidial

Fumagillin (**21**) was isolated from the culture broth of the fungus *Aspergillus fumigatus* as an antiphage and amebicidal antibiotic.52) It was used to control microsporidial disease due to *Nosema apis* in honeybees for more than 40 years.44) Microsporidia are also responsible for opportunistic infections in patients with AIDS. Recently, fumagillin has been used topically to treat ocular microsporidiosis caused by *Encephalitozoon hellem* or *E. intestinalis*. 53) Antiangiogenic activity of fumagillin is also known, and its target was revealed as methionine aminopeptidase 2 (MetAP2).54) Since microsporidia appear to lack MetAP1 and to carry only the gene for MetAP2, fumagillin should be particularly promising because the absence of MetAP1 would prevent microsporidia from compensating for the loss of MetAP2 activity when exposed to fumagillin.55)

## Anthelmintics

Helminths include nematodes (roundworms), trematodes (flukes), and cestodes (tapeworms) as shown in Fig. 1. Nematodes have a cylindrical or filamentous shape and belong to the phylum Aschelminthes. Trematodes and cestodes have a flat shape and belong to the phylum Platyhelminthes. Trematodes have a digestive tract, but cestodes do not, and are composed of many segments. We describe some important and interesting anthelmintic antibiotics below.

### (a) Avermectins and milbemycins

Avermectins and milbemycins are nematocidal and insecticidal antibiotics. They are spiro-ketal 16-membered ring macrolides. Avermectins have a sugar moiety at C-13, but milbemycins have no hydroxyl group at C-13 and therefore have no sugar moiety. Eight components of avermectins were isolated from the culture broth of *Streptomyces avermectinius*56) by cooperative research between our group and a research group at Merck Sharp & Dohme Research Laboratories, USA, and they show potent anthelmintic activities against many nematodes.57),58) Recently, our group elucidated the complete genome sequence of *S. avermectinius*.59),60) The genome contains 9,026 kb, which is the largest bacterial genome yet sequenced, and it encodes at least 7,574 potential open reading frames. It is interesting that strains producing milbemycin-type compounds are found commonly among *Streptomyces* spp., but strains of *S. avermectinius* that produce avermectin-type compounds are rare.61) Although only one additional *S. avermectinius* strain, isolated in Italy, has ever been reported,62) no novel avermectin was isolated from this strain. About 40 avermectin homologs produced by mutants of *S. avermectinius* and more than 100 milbemycin homologs produced by *Streptomyces* spp. or their mutants have been reported.

**Fig. 5:**
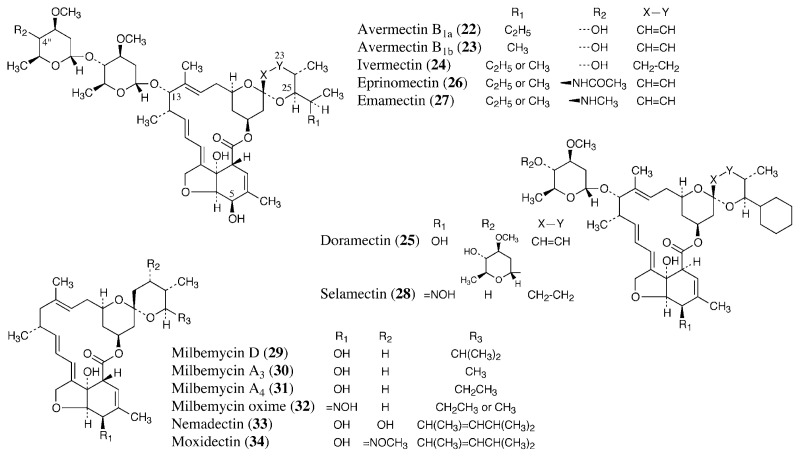
Avermectins and milbemycins.

Avermectin B_1_ is composed of avermectin B_1a_ (**22**, Fig. 5) and avermectin B_1b_ (**23**), and ivermectin (**24**), a mixture of 22,23-dihydro derivatives of **22** and **23**, has a greater safety profile than the original mixture of compounds. 63) Ivermectin is used for antiparasitic control of the economically important gastrointestinal, lung, and kidney nematodes, as well as other nematodes such as *Thelazia* and *Parafilaria* in livestock.61) It is also used against a number of arthropod parasites, including grubs, lice, mites, and screw worms. In companion animals, it provides highly effective control of *Dirofilaria immitis* (heartworm).

Ivermectin is also used clinically for controlling some human nematode infections. *Onchocerca volvulus* causes human onchocerciasis (river blindness), an infection characterized by chronic skin and eye lesions. Ivermectin is highly efficacious against the microfilariae of *O. volvulus* and is used in programs dedicated to controlling onchocerciasis in the endemic areas of Africa and the Americas.64) Recently, it has also become an invaluable tool for a control program against lymphatic filariasis (elephantiasis) caused by *Wuchereria bancrofti* and *Brugia malayi*.65) Strongyloidiasis, distributed in the tropics and subtropics, is an intestinal disease caused by the nematode *Strongyloides stercoralis*. Ivermectin is currently the most useful agent against *S. stercoralis*.66) In addition to the control of nematode infections, ivermectin shows efficacy for human scabies, a skin disease caused by infection with the ectoparasitic mite *Sarcoptes scabiei*.

Avermectin B_1_, under the non-proprietary name abamectin, is used as a gastrointestinal nematocide through subcutaneous injection for cattle and also as an agricultural miticide.61) Doramectin (**25**) is a C-25 substituted avermectin B_1_ produced by mutational biosynthesis. 67) Cyclohexane carboxylic acid was fed to a mutant strain of *S. avermectinius* lacking branched-chain 2-oxo acid dehydrogenase activity. Doramectin was developed for uses similar to those of ivermectin in the veterinary field. The other semisynthetic avermectins are commercially available as nematocides or insecticides. 68) Eprinomectin (**26**), a 4″-epi-(acetylamino)-4″-deoxy derivative of avermectin B_1_, is used topically for control of gastrointestinal and the other parasitic nematodes and a number of arthropods in cattle, including lactating cows. It possesses one of the lowest milk partitioning coefficients in this class of antiparasitics. Emamectin (**27**), a 4″-epi-(methylamino)-4″-deoxy derivative of avermectin B_1_, is used as an agricultural insecticide, particularly against the order Lepidoptera. Selamectin (**28**), a 5-oxoimino derivative of doramectin monosaccharide, is used to control nematodes and fleas in cats and dogs.

Milbemycins are originally isolated from the culture broth of *Streptomyces hycroscopicus*.69) Milbemycin D (**29**) is used as a nematocide in the veterinary field, and milbemectin, a mixture of milbemycins A_3_ (**30**) and A_4_ (**31**), is used as an agricultural acaricide.68) Milbemycin oxime (**32**), a 5-oxoimino derivative of milbemectin, has been launched as a nematocide for dogs. Nemadectin (**33**) is a milbemycin group antibiotic produced by *Streptomyces cyaneogriseus*.70) Moxidectin (**34**) is a 23-methyloxime derivative of nemadectin, used for control of nematodes and arthropods in the veterinary field.

The major target of avermectins is a glutamate-gated chloride channel, which does not exist in vertebrates. 71) Avermectins activate the channel, causing neurological effects. Although avermectins also bind to GABA- and glycine-gated chloride channels in mammals, their affinity for invertebrate receptors is approximately 100-fold higher. Knock-out mice of the P-glycoprotein gene showed increased sensitivity to ivermectin.72) This result established an important role for the P-glycoprotein in the maintenance of blood-brain barrier, and suggests that the safety of avermectins is due to the blood-brain barrier, dependent on P-glycoprotein, in addition to the low affinity of the receptors.

**Fig. 6:**
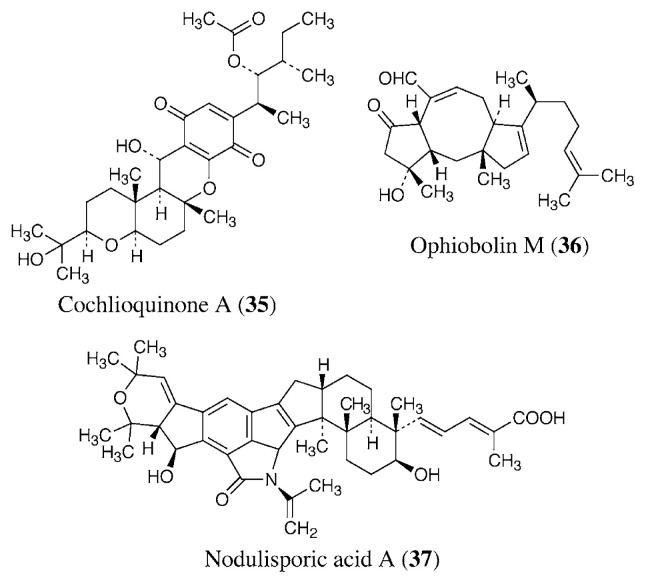
Inhibitors of ivermectin binding to its binding site.

The fungal toxins cochlioquinone A (**35**, Fig. 6) and ophiobolins were isolated as nematocidal compounds. Cochlioquinone A, produced by the fungi *Cochliobolus miyabeanus* and *Helminthosporium sativum*, showed competitive inhibition against [^3^H]ivermectin binding to membranes prepared from the free-living nematode *Caenorhabditis elegans*.73) On the other hand, ophiobolin M (**36**) and its analogs, produced by *Bipolaris* spp. fungi, are noncompetitive inhibitors of ivermectin binding.74) It has been suggested that cochlioquinone A and ophiobolins may have a similar target to ivermectin. Recently, a potent insecticidal antibiotic, nodulisporic acid A (**37**), and its analogs were isolated from the culture broth of a endophytic fungus, *Nodulisporium* sp.75) Nodulisporic acid A activates a glutamate-gated chloride channel like ivermectin, but lacks activity at GABA- and glycine-gated chloride channels. However, no useful anthelmintic activity was observed *in vitro* or *in vivo*.76)

### (b) Aminoglycosides

**Fig. 7:**
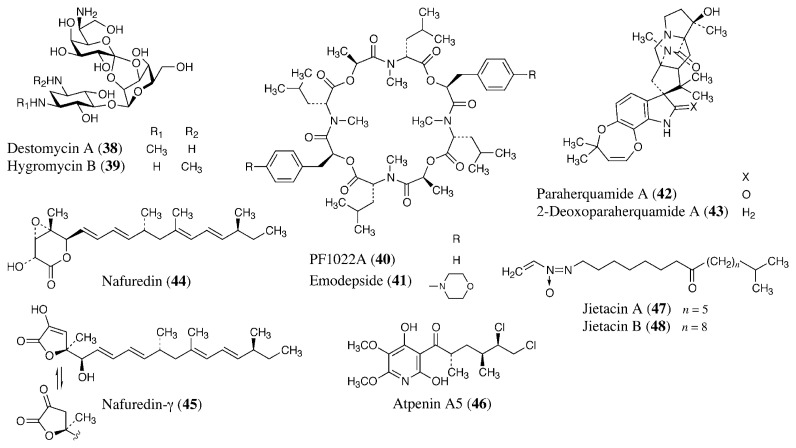
Anthelmintic antibiotics.

Aminoglycoside antibiotics are protein synthesis inhibitors, and some of them are used as anthelmintics. Paromomycin (**16**) is used for treatment of human and animal cestode infections.77) It is also useful for some protozoan infections as mentioned above. Destomycin A (**38**, Fig. 7) and hygromycin B (**39**) are produced by *Streptomyces rimofaciens* and *S. hygroscopicus*, respectively.78),79) They are aminoglycosides used as feed additives for treatment of nematode infections by *Ascaris*, *Oesophagostomum*, *Heterakis*, *Trichuris*, and *Capillaria* in the veterinary field.80) Our group found that hygromycin A coproduced with **39** shows good therapeutic effect for swine dysentery caused by a spirochete *Treponema hyodysenteriae*. 81),82)

### (c) Emodepside

PF1022A (**40**) is a cyclic octadepsipeptide nematocide produced by a fungus, *Rosellinia* sp.83)–85) Emodepside (BAY 44-4400, **41**) is a semisynthetic derivative of PF1022A with a morpholine ring at each of the two d-phenyllactic acids in the para position. Its target was identified as a novel 110 kDa heptahelical transmembrane receptor, named HC-110R, which is similar to mammalian latrophilins.86) Latrophilins are latrotoxin (black widow spider venom) receptors and G protein-coupled receptors implicated in the regulation of exocytosis. Latrotoxin also binds to HC110-R and causes influx of external Ca^2+^. Emodepside acts as an antagonist to latrotoxin signaling by impairing the influx of Ca^2+^. Emodepside exerts a high efficacy against a wide variety of nematodes in different animal species, and it possesses resistance-breaking properties against the usual classes of anthelmintics.84) It will be commercially available as a veterinary nematocide soon.

### (d) Paraherquamide group compounds

Brevianamides, paraherquamides, and marcfortines are a group of oxindole alkaloids produced by *Penicillium* spp. fungi.87)–89) Their nematocidal activity was initially detected in a gerbil and *Trichostrongylus colubriformis* model using paraherquamide A (**42**).90) A semisynthetic analog, 2-deoxoparaherquamide A (PNU-141962, **43**), has an excellent nematocidal activity and safety profile, and is currently under development.91) Although they are nematocidals, they do not have insecticidal activity. They are antagonists of nicotinic acetylcholine receptors and block cholinergic neuromuscular transmission.92) There are differences between the sensitivity of nematodes and mammals to paraherquamides due to intrinsic differences in receptor affinity between these phyla.

### (e) Nafuredin

**Fig. 8:**
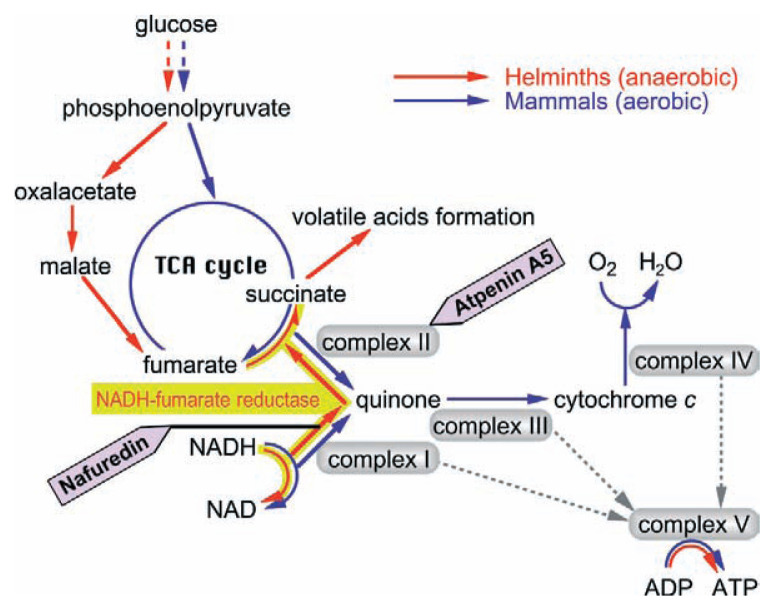
Energy metabolism in helminths and mammals.

Differences in energy metabolisms between the host and helminths are attractive targets for treatment of helminthiasis (Fig. 8). The NADH-fumarate reductase system is part of a special respiratory system in parasitic helminths, which is found in many anaerobic organisms. The system is composed of complex I (NADH-rhodoquinone oxidoreductase) and complex II (rhodoquinol-fumarate reductase). Electrons from NADH are accepted by rhodoquinone through complex I, and then transferred to fumarate through complex II (Fig. 7). This anaerobic electron transport system can provide ATP in the absence of oxygen. We screened for inhibitors of NADH-fumarate reductase using *Ascaris suum* (roundworm) mitochondria, and obtained a novel antibiotic, nafuredin (**44**), produced by an *Aspergillus niger* strain isolated from a marine sponge.93)–95)

Nafuredin inhibits NADH-fumarate reductase and NADH-rhodoquinone oxidoreductase of *A. suum* at nanomolar concentrations, while it shows only weak inhibition of rhodoquinol-fumarate reductase (Table II). Therefore, nafuredin is a complex I inhibitor. However, it inhibits rat liver complex I (NADH-ubiquinone oxidoreductase) at very high concentration. It is interesting that nafuredin inhibits not only anaerobic adult complex I of *A. suum* but also larval complex I, which has an aerobic energy metabolism like that of mammals. Thus, nafuredin is a selective inhibitor of helminth complex I.

Nafuredin shows anthelmintic activity against *Haemonchus contortus* (barberpole worm) in *in vivo* trials using sheep. This anthelmintic activity may be due to the inhibition of complex I because nafuredin also inhibits the enzyme of *H. contortus* (Table II). Nafuredin is a novel anthelmintic lead compound, and we have accomplished its total synthesis.96) Recently, we found that nafuredin is converted to a novel *γ*-lactone derivative named nafuredin-*γ* (**45**) under mild basic conditions, and this derivative has the same enzyme inhibitory and anthelmintic activity. Since the lactone moiety synthesis of nafuredin-*γ* is simpler than that of nafuredin, nafuredin-*γ* is useful for the structure-activity relationship study. We have also achieved its total synthesis and are studying its analogs.97)

In the same screening, we rediscovered atpenin A5 (**46**) and related compounds as NADH-fumarate reductase inhibitors. Atpenins were originally isolated from the culture broth of *Penicillium* sp. as antifungal antibiotics by our group.98) In contrast to nafuredin, atpenins inhibit complex II (succinate-ubiquinone oxidoreductase), and the inhibition is nonselective between helminths and mammals.99) Although there are potent inhibitors of complexes I, III, and IV, no very potent inhibitors of complex II have been described. The IC_50_ value of atpenin A5 is 300-fold lower than that for carboxin, the most potent known complex II inhibitor. Therefore, atpenins may be useful tools for clarifying the biochemical and structural properties of complex II. Recently, the X-ray crystallographic structure of *Escherichia coli* complex II was elucidated.100) Eukaryotic helminth and mammalian complex II structures may be determined soon. This information may clarify the interaction of atpenins and complex II, and give a clue to design of helminth-specific atpenin analogs.

### (f) Other anthelmintics

Avermectin and milbemycin group macrolides are used as nematocides as described above. Some other macrolides are also reported to have nematocidal activities. We showed that the 26-membered ring macrolides, oligomycins A and G, produced by *Streptomyces* sp. have nematocidal activity *in vitro*, but they are also cytotoxic.101) Oligomycins are complex V (H^+^-transporting ATP synthase) inhibitors. A 14-membered ring macrodiolide, clonostachydiol, produced by the fungus *Clonostachys cylindrospora*, and a 16-membered ring macrodiolide, pyrenophorol (helmidiol), produced by some fungi were active against *Haemonchus contortus in vivo*.102),103) Axenomycins are 34-membered ring macrolides with sugar and naphthoquinone moieties produced by *Streptomyces lysandri*. They show *in vivo* cestocidal activity.104),105)

Jietacins A (**47**) and B (**48**), which were isolated from the culture broth of *Streptomyces* sp. by our group, have unique vinylazoxy moieties.106) They were isolated based on screening using the pine wood nematode *Bursaphelenchus lignicolus*,107) and they show ten times higher nematocidal activity than avermectin B_1a_ against *B. lignicolus in vitro*.

Antiparasitic antibiotics with a variety of structures have been discovered by the efforts of many researchers, mainly as metabolites of actinomycetes and fungi. Our group alone has isolated about fifty new antibiotics. These compounds affect various biological targets, such as protein synthesis, energy metabolism, lipid metabolism, neurotransmitters, and cellular integrity, and have selectivity against susceptible parasites. Although not many antiparasitic antibiotics are currently used clinically, some are commercially available as tools for biochemical studies. We hope that further work in this field shall yield effective antiparasitic remedies.

## Figures and Tables

**Table I:** Practically used antiparasitic antibiotics

Antibiotics	Producing strain	Disease	Mode of action
**Antiprotozoals**			
Tetracycline (**1**)	*Streptomyces* spp.	malaria	protein synthesis inhibitor
Polyethers (**10**)	actinomycetes	coccidioisis	increase the influx of cations
Spiramycin (**13**)	*S. ambofaciens*	cryptosporidiosis	protein synthesis inhibitor
Acetylspiramycin (**14**)	semisynthetic	toxoplasmosis	protein synthesis inhibitor
Clindamycin (**15**)	semisynthetic	toxoplasmosis & babesiosis	protein synthesis inhibitor
Paromomycin (**16**)	*S. rimosus*	cryptosporidiosis, amebiasis & giardiasis	protein synthesis inhibitor
Amphotericin B (**17**)	*S. nodosus*	leishmaniasis	bind to membrane
Trichomycin (**19**)	*S. hachijoensis*	trichomoniasis	bind to membrane
Fumagillin (**21**)	*Aspergillus fumigatus*	microsporidiasis	Met aminopeptidase 2 inhibitor
**Anthelmintics**			
Ivermectin (**24**)	semisynthetic	nematode infections	Glu-gated Cl^−^ channel agonist
Milbemycin D (**29**)	*S. hycroscopicus*	nematode infections	Glu-gated Cl^−^ channel agonist
Destomycin A (**38**)	*S. rimofaciens*	nematode infections	protein synthesis inhibitor
Hygromycin B (**39**)	*S. hygroscopicus*	nematode infections	protein synthesis inhibitor
Paromomycin (**16**)	*S. rimosus*	cestode infections	protein synthesis inhibitor

**Table II:** Effects of nafuredin on electron transport enzymes of *Ascaris suum*, *Haemonchus contortus*, and rat liver

	Complex	IC_50_ (nM)				

		*A. suum* (adult)	*A. suum* (L2)	*H. contortus* (adult)	*H. contortus* (L3)	Rat liver
NADH-fumarate reductase	I+II	12	[Table-fn tfn1-pjab-80-245]–	[Table-fn tfn1-pjab-80-245]–	[Table-fn tfn1-pjab-80-245]–	1,000
NADH-ubiquinone reductase	I	8	8.9	86	120	10,000
NADH-rhodoquinone reductase	I	24	9.0	195	350	>100,000
Rhodoquinol-fumarate reductase	II	80,000	[Table-fn tfn1-pjab-80-245]–	[Table-fn tfn1-pjab-80-245]–	[Table-fn tfn1-pjab-80-245]–	[Table-fn tfn1-pjab-80-245]–
Succinate-ubiquinone reductase	II	>100,000	[Table-fn tfn1-pjab-80-245]–	[Table-fn tfn1-pjab-80-245]–	[Table-fn tfn1-pjab-80-245]–	>100,000

– : not tested.
